# Effect of Epigallocatechin‐3‐Gallate on Depression‐Related Cytokines in Thalassemia Patients: Molecular and Cellular Evaluation

**DOI:** 10.1002/jcla.70171

**Published:** 2026-01-28

**Authors:** Mohammed N. Salman, Fouad Razzaq Al‐Burki, Hazim Ali Hussein, Laith A. Younus, Fadhil A. Nasser, Hasanain A. A. Almohseni

**Affiliations:** ^1^ Department of Clinical Laboratory Sciences, College of Pharmacy Jabir Ibn Hayyan University for Medical and Pharmaceutical Sciences Kufa Iraq; ^2^ Department of Pharmacognosy, College of Pharmacy Jabir Ibn Hayyan University for Medical and Pharmaceutical Sciences Kufa Iraq; ^3^ Department of Clinical Pharmacy, College of Pharmacy Jabir Ibn Hayyan University for Medical and Pharmaceutical Sciences Kufa Iraq; ^4^ Department of Pharmaceutical Chemistry, College of Pharmacy Jabir Ibn Hayyan University for Medical and Pharmaceutical Sciences Kufa Iraq

**Keywords:** cytokines, epigallocatechin, gene, thalassemia, Winged Marigold

## Abstract

**Background:**

Epigallocatechin‐3‐gallate (EGCG) is the major polyphenolic compound found in Winged Marigold and Green tea. It exhibits well‐established anti‐inflammatory and antioxidant characteristics. EGCG has been shown to suppress the expression of several pro‐inflammatory cytokines, including IL‐6, IL‐1β, TNF‐α, and IFN‐γ. However, its effect on inflammation‐related cytokines associated with depression in β‐thalassemia patients remains incompletely understood.

**Methods:**

Five peripheral blood mononuclear cell (PBMC) samples from β‐thalassemia patients were selected for this study in order to demonstrate how EGCG affects the inflammatory state in thalassemic individuals. EGCG was extracted from Winged Marigold using an ethanol‐based method, and its purity was confirmed using HPLC and LC–MS/MS analyses. PBMCs were treated with ethanolic solvent alone (control) or with EGCG at concentrations of 5, 25, and 50 μM. Cell viability was assessed and compared with untreated controls, and cytokine gene expression was evaluated using RT‐qPCR.

**Results:**

EGCG exhibited a statistically significant cytotoxic effect at concentrations above 10 μM (*p* < 0.005), with highly significant effects observed at 25 and 50 μM (*p* < 0.001). Increasing EGCG concentrations up to 50 μM resulted in a significant reduction in cytokine gene expression, with *p*‐values ranging from < 0.001 to < 0.05.

**Conclusion:**

EGCG significantly reduces the expression of depression‐related inflammatory cytokines in PBMCs derived from β‐thalassemia patients. These findings suggest that EGCG may have a potential modulatory role in inflammatory pathways associated with depression in thalassemia, although dose‐dependent cytotoxic effects should be carefully considered.

## Graphical abstract

This study investigates the effects of Epigallocatechin‐3‐Gallate (EGCG), a potent anti‐inflammatory polyphenol isolated from Winged Marigold, on cytokine expression linked to depression in β‐thalassemia patients. EGCG demonstrated dose‐dependent suppression of key inflammatory cytokines (IL‐6, IL‐1β, TNF‐α, IFN‐γ) at both gene and protein levels, suggesting its potential as an adjunctive therapy to alleviate neuroinflammatory symptoms associated with thalassemia.

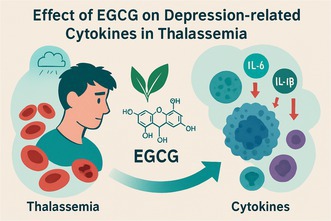

## 1 Background

Defects in hemoglobin synthesis or protein structure result in hemoglobinopathies. The most common hemoglobinopathy is thalassemia, with an estimated 5% of people worldwide having at least one thalassemia variant allele [1].

Alpha and beta thalassemia are the two varieties. Individuals with beta thalassemia can present with hypochromic microcytic anemia with elevated HbA2 or as silent carriers with acceptable hematological markers. However, individuals with beta thalassemia major and intermedia are known as transfusion‐dependent or non‐transfusion‐dependent thalassemia, respectively, and need transfusions either regularly or infrequently. From transfusion‐dependent patients who may potentially develop multi‐organ problems to asymptomatic individuals, the clinical picture of β‐thalassemia varies greatly [2].

The management of β‐thalassemia is fraught with challenges. Traditional treatments such as regular transfusions, iron chelation therapy, and splenectomy can lead to systemic complications. These protocols and treatment tools sometimes could lead to increased oxidative stress, continuous inflammation, and disruption of immune system regulation. From an immunological point of view, many patients show abnormalities in T and B lymphocyte subtypes, higher white blood cell counts, and altered cytokine secretion profiles [3].

Documented immunological studies have shown specific alterations in T cell populations in these patients, where reduced CD4^+^ helper T cells and elevated CD8^+^ cytotoxic T cells lead to a decreased CD4/CD8 ratio. This imbalance is correlated with decreased proliferation of lymphocytes and decreased function of natural killer (NK) cells; as a result, there is increased susceptibility to infections [4].

Many researchers are now using a novel method to investigate the relationship between immunological dysregulation and thalassemia‐related mental decline. Anxiety and depression are among the psychological conditions linked to chronic systemic inflammation. Immune activity and mental health are biologically related because the development and progression of depressive signs and symptoms have been associated with increased expression of pro‐inflammatory cytokines such as interleukin‐6 (IL‐6), interleukin‐1β (IL‐1β), tumor necrosis factor‐alpha (TNF‐α), and interferon‐gamma (IFN‐γ). Additionally, these pro‐inflammatory cytokines interfere with neural plasticity, neurotransmitter balance, and hypothalamic–pituitary–adrenal (HPA) axis control [5].

Epigallocatechin‐3‐Gallate (EGCG), a bioactive polyphenol found in green tea, is also found in other plants such as the Winged Marigold [6].

Additionally, and due to its anti‐inflammatory, antioxidant, and neuroprotective properties, EGCG has been widely studied. Various inflammatory cytokines are suppressed, many free radicals are neutralized, and key inflammatory signaling sequelae, including the nuclear factor kappa‐light‐chain‐enhancer of activated B cells (NF‐κB) pathway, are inhibited by EGCG.

Due to the immune modulating properties of EGCG, it could be a promising medication for alleviating neuroinflammatory symptoms associated with β‐thalassemia [7]. Till now, there has been a noticeable lack of studies examining the effects at both the molecular and cellular levels. Techniques such as reverse transcription quantitative polymerase chain reaction (RT‐qPCR) for expression of gene, in addition to ELISA and 3‐(4,5‐dimethylthiazol‐2‐yl)‐2,5‐diphenyltetrazolium bromide (MTT) assays for protein expression and cellular function, represent a great chance to explore the activity of this herbal medication [8].

By employing both molecular and cellular methodologies, the present research aimed to evaluate the effects of EGCG on the expression of cytokines associated with depression in β‐thalassemia patients. The study aimed to provide a more complete understanding of the therapeutic mechanisms of EGCG. The findings made could possibly permit the development of adjunctive therapies designed to address both the psychological and immunological dimensions of β‐thalassemia.

## 2 Materials and Methods

### 2.1 GC MS Analysis

*Extraction*: Using 75% ethanol as a solvent, the extraction procedures were performed in accordance with Harborne's [9] technique to extract the active chemicals. Then, using a gas chromatography‐mass spectrometer (GC‐MASS) (Agilent 5977 A MSD, USA), the number of active compounds in the plant leaves was calculated, utilizing the Mass Hunter GC/MS Acquisition and Mass Hunter qualitative software (USA). All procedures were performed in accordance with the manufacturer's guidelines.

### 2.2 Isolation of Epigallocatechin‐3‐Gallate

To isolate EGCG from the plant extract, the standard method of Silica Gel Column Chromatography (SGC) described by Amarowicz et al. [10] was used, with fraction detection via TLC. The target compound was then purified using semi‐preparative and analytical RP C18 HPLC, according to the steps described by Bhatia et al. [11], Blahová and Lehotay [12].

### 2.3 Purity and Identity Confirmation of Isolated EGCG

#### 2.3.1 Analytical HPLC Procedure

The isolated compound was analyzed using HPLC to ensure its purity according to the method described by Tra and Bui [13]. An Agilent 1260 Infinity was used (column: C18: 250 × 4.6 mm, 5 μm). The mobile phase was a gradient extraction of solvent A: water with 0.1% trifluoroacetic acid (TFA) and solvent B: acetonitrile. The gradient was from 10% to 40% of TFA for the required time (20 min). The flow rate was 1.0 mL/min. At a wavelength of 280 nm UV light was used for detection. The injection volume was 20 μL and the retention time (RT) for the standard EGCG was ≈11.8 min, while the retention time for the isolated compound was ≈11.8 min (Table 1).

**TABLE 1:** HPLC parameters and retention times.

% Sample	RT (min)	Peak area	Peaks number	Purity
EGCG standard	11.81	100%	1	100.00%
Isolated EGCG	11.86	98.70%	1	98.78%

**FIGURE 1:**
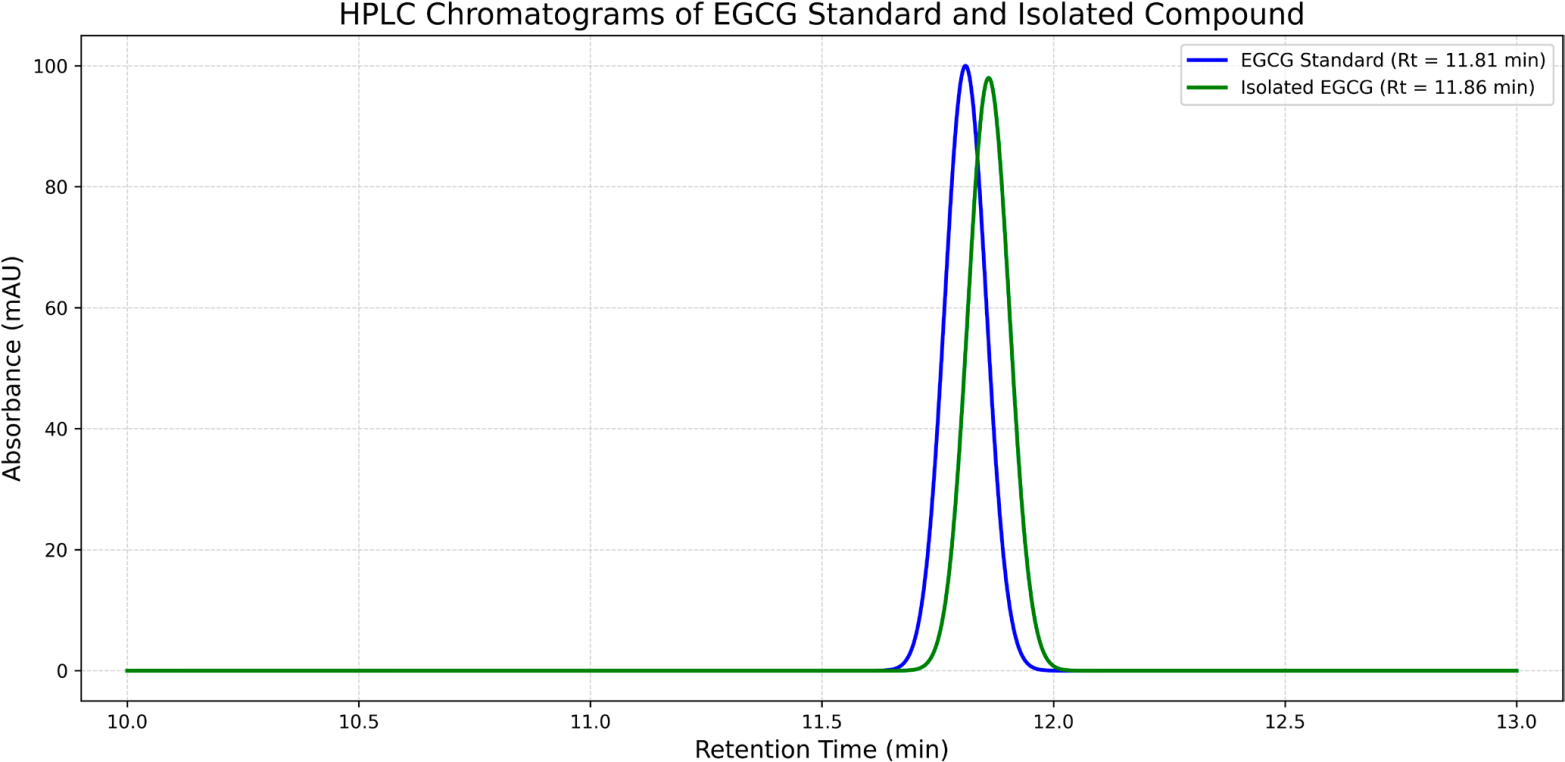
HPLC chromatograms results.

Two sharp peaks, one at 11.81 min (the standard) and the other at 11.86 min (representing the isolated sample) were noted in the graph (Figure 1). The similarity of the two peaks in terms of shape and location confirmed the success of the isolation with a high purity rate of 98.78% (Figure 1).

### 2.4 LC–MS/MS Analysis

**FIGURE 2:**
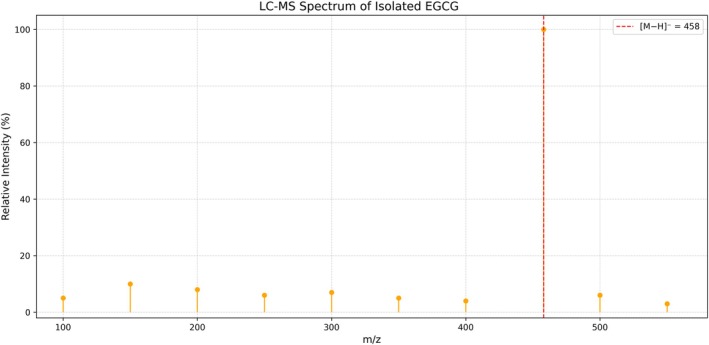
LC–MS spectrum of the isolated sample of EGCG.

To confirm the molecular identity of the isolated EGCG according to the method described by Niessen [14], mass spectrometry analysis was performed accordingly. The ionization mode was negative ESI, with predicted mass/energy of 457 [M−H]^−^ and detected mass/energy of 457.09. The molecular ion peak matched exactly with the theoretical mass of EGCG (C_22_H_18_O_11_, MW = 458.37), confirming the identity (Figure 2).

Figure 2 shows the main peak (red) at *m/z* = 458.37, representing the [M − H]^−^ ion of Epigallocatechin‐3‐Gallate, confirming the identity of the EGCG compound.

### 2.5 Cell Viability Assay (MTT Assay)

To identify the cytotoxic activity of an ethanolic extract of Winged Marigold, containing purified Epigallocatechin‐3‐Gallate (EGCG), an MTT assay was performed (at Al‐Amin Center for Advanced Biotechnology, Najaf, in collaboration with the Cancer Research Center at the University of Tehran) on β‐thalassemia patients' peripheral blood mononuclear cells (PBMCs), and all the steps according to the protocol described by Mosmann [15] were carried out.

### 2.6 Cell Culture and Treatment

To extract PBMCs from whole blood samples by methods described by Boyum [16], Ficoll–Hypaque density gradient centrifugation was utilized. Prior to being incubated at 37°C, they were cultivated at a density of 1 × 105 cells/well in RPMI‐1640 media (Gibco, Thermo Fisher Scientific, USA) supplemented with 10% fetal bovine serum and 1% penicillin–streptomycin. PBMCs were subsequently exposed to EGCG at concentrations of 5, 25, or 50 μM for a duration of 24 h. Two control groups were also included, one of which was left untreated and the other of which was given an equal ethanolic solvent.

### 2.7 MTT Assay Procedure

Following the 24‐h treatment period referred to above, 10 μL of MTT solution (5 mg/mL) was added to each well. The wells were then incubated for 4 h at 37°C. The formazan crystals were then placed in 100 μL of dimethyl sulfoxide (DMSO) solution, and the absorbance was finally measured at 570 nm by using a microplate reader. The percentage of cells that survived treatment was compared to that in the untreated control. Every experiment was performed in triplicate.

### 2.8 Statistical Analysis

Standard deviation (SD) ± mean was used to represent the data. To identify pairwise differences, one‐way Tukey's post hoc test was used. Analysis of Variance (ANOVA) was used to evaluate statistical significance between groups. To conduct statistical studies, GraphPad Prism version 9.0 (GraphPad Software, San Diego, CA, USA) was utilized. At *p* < 0.05, differences were deemed statistically significant.

### 2.9 RT‐qPCR for Cytokine Gene Expression

Quantitative real‐time PCR (RT‐qPCR) was performed on RNA extracted from treated PBMCs to assess the regulatory effect of purified Epigallocatechin‐3‐Gallate (EGCG) on depression‐related cytokine gene expression in β‐thalassemia patients.

### 2.10 Treatment and Design of Experiments

As previously mentioned, PBMCs were isolated and cultivated. Untreated, ethanol‐equivalent, and EGCG‐treated groups were created. In the EGCG‐treated group, EGCG was added to the cells at concentrations of 5 and 25 μM. The MTT assay results, which showed non‐toxic and physiologically effective doses, served as the basis for the treatment concentrations that were chosen. The treatments were incubated with the cells for a full day.

### 2.11 Total RNA Extraction and cDNA Synthesis

By using the TRIzol reagent (Invitrogen, USA), total RNA was extracted following the manufacturer's protocol. The purity and concentration of RNA were assessed by Nano drop spectrophotometry (260/280 ratio).

In a 20 μL reaction volume one microgram of RNA was reverse‐transcribed to cDNA using the Revert Aid First Strand cDNA Synthesis Kit (Thermo Fisher Scientific).

By using Primer‐BLAST from the National Center for Biotechnology Information (NCBI) website (https://www.ncbi.nlm.nih.gov/), the Primers for the five genes under study were designed as in (Tables 2 and 3).

**TABLE 2:** Primers sequences for cytokine genes and standard conditions.

Gene	Primer sequences	Annealing temp./Time
IL‐6	F: 5′AGACAGCCACTCACCTCTTCAG3′ R: 5′TTCTGCCAGTGCCTCTTTGCTG3′	60°C/60 s
TNF‐α	F: 5′CCTCTCTCTAATCAGCCCTCTG3′ R: 5′GAGGACCTGGGAGTAGATGAG3′	
IL‐1β	F: 5′ATGATGGCTTATTACAGTGGCA3′ R: 5′GTCGGAGATTCGTAGCTGGA3′	
IFN‐γ	F: 5′TGGAAAGAGGAGAGTGACAG3′ R: 5′GCTCTGCAGGATTTTCATGTC3′	
GAPDH	F: 5′GAAGGTGAAGGTCGGAGTC3′ R: 5′GAAGATGGTGATGGGATTTC3′	

*Note:* Initial denaturation for 40 cycles at 95°C for 10 min—15 s for denaturation at 95°C—Annealing/Extension at 60°C for 60 s.

**TABLE 3:** The RT‐qPCR reaction included the following.

Material name	Volume (μL)
Master Mix of SYBR Green	10
Primer forward	0.5
Primer reverse	0.5
cDNA	2
Water (nuclease‐free)	7
Total	20

### 2.12 Data Analysis

Relative gene expression was calculated using the 2^‐ΔΔCt method, normalized to GAPDH, and expressed as fold change relative to the control group: −ΔCt = Ct (BCL2) − Ct (GAPDH).

$-\text{ΔΔCt}=\mathrm{\Delta Ct}\;\left(\text{treatment}\right)-\mathrm{\Delta Ct}\;\left(\text{control}\right)$

All reactions were performed in triplicates (*n* = 3). Three duplicates of each reaction were carried out. For statistical analysis, One‐way ANOVA test and Tukey's post hoc test were used, *p* < 0.05 considered statistically significant.

## 3 Results and Discussion

### 3.1 GC MS Analysis of Winged Marigold Extract

The results of the GC MS analysis of the Winged Marigold leaf extract, shown in Table 4, revealed the presence of 34 compounds belonging to many chemical classes, most notably phenols, nitrogenous cyclic compounds (morpholine and pyrrolidine), and sulfur compounds (dimethyl trisulfide), in addition to esters and organic acids. Epigallocatechin‐3‐Gallate (EGCG) constituted the highest percentage (25.02%), reinforcing its importance as a key compound that may contribute to biological activity associated with cytokine modulation. Other phenolic compounds, such as Catechol and 4‐Vinylphenol, were also present; these are known for their antioxidant properties and potential anti‐inflammatory effects.

**TABLE 4:** Active phytochemicals in the leaves of the Winged Marigold.

Peak	R.T.	Area Pct%	Library/ID	Ref
1	5.391	2.9994	Pyrrolidine	692
2	6.585	3.307	Morpholine	2090
3	7.669	0.3719	Dimethyl Sulfoxide	1144
4	8.659	0.3204	2‐Methyl‐1‐vinylimidazole	6015
5	9.547	0.6894	3H‐Pyrazol‐3‐one, 2,4‐dihydro‐4,4,5‐trimethyl—	12,673
6	9.657	4.9475	Dimethyl trisulfide	12,503
7	9.955	1.0397	1,5‐Hexadiene, 3‐chloro—	9433
8	10.152	0.2815	dl‐Threitol	11,035
9	10.411	0.4244	Hydroxyurea, N, N′, O‐trimethyl—	9807
10	10.631	0.2723	dl‐Threitol	11,035
11	10.961	0.5425	1‐(1′‐Pyrrolidinyl)‐2‐propanone	13,576
12	11.652	0.3287	2,5‐Dimethylfuran‐3,4(2H,5H)‐Dione	13,925
13	11.825	0.9743	2‐Pyrrolidinone	1737
14	12.225	0.6675	But‐3‐enyl (E)‐2‐methylbut‐2‐enoate	32,167
15	12.312	0.7729	Pyridine, 2,3,4,5‐tetrahydro—	1484
16	12.414	0.9985	Succinic acid, 1‐methoxydec‐4‐yl pentyl ester	267,896
17	12.72	0.2788	1‐Piperidinamine	4075
18	12.893	0.472	Ethanamine, N‐ethyl‐N‐nitroso—	4735
19	13.027	1.7945	4H‐Pyran‐4‐one, 2,3‐dihydro‐3,5‐dihydroxy‐6‐methyl—	23,823
20	13.459	0.3349	N‐(4‐Aminophenyl)‐N‐methyl‐2‐(4‐methylpiperazin‐1‐yl) acetamide	150,503
21	13.891	0.5067	Catechol	6512
22	14.009	0.2916	1‐azetidinepropanoic acid, ethyl ester	34,373
23	14.119	0.4911	4‐Vinylphenol	10,651
24	14.551	0.8568	1‐Methyl‐2‐pyrrolidineethanol	14,884
25	14.888	0.6483	1,2,2‐Trimethylpropyl trifluoroacetate	74,308
26	15.532	1.0929	2‐Methoxy‐4‐vinylphenol	28,303
27	15.58	0.973	Tropidine, 2‐acetyl‐8‐demethyl—	28,964
28	15.878	0.304	5‐Hexenal, 4‐methylene—	6576
29	15.933	0.4327	Phthalic acid, ethyl 3,4‐dimethylphenyl ester	197,021
30	16.271	0.3285	2,8,9‐Trioxa‐5‐aza‐1‐silabicyclo (3.3.3) undecane, 1‐methoxy—	81,701
31	16.452	0.2752	N‐Nitroso‐2,4,4‐trimethyloxazolidine	23,715
32	16.891	25.0229	Epigallocatechin‐3‐Gallate	27,157
33	17.221	0.6002	Decane, 5,6‐dimethyl—	45,510
34	17.944	1.0329	N‐(4‐Chlorobenzylidene) methylamine	30,805

### 3.2 MTT Assay

Epigallocatechin‐3‐Gallate (EGCG), isolated from Winged Marigold extract, showed a dose‐dependent concentration effect on immune cells (PBMCs) isolated from thalassemia patients. The Control and Vehicle groups revealed high cell survival rates (100.0% and 98.7%, respectively), suggesting that the Vehicle does not have any toxic effect per se (not significant).

**FIGURE 3:**
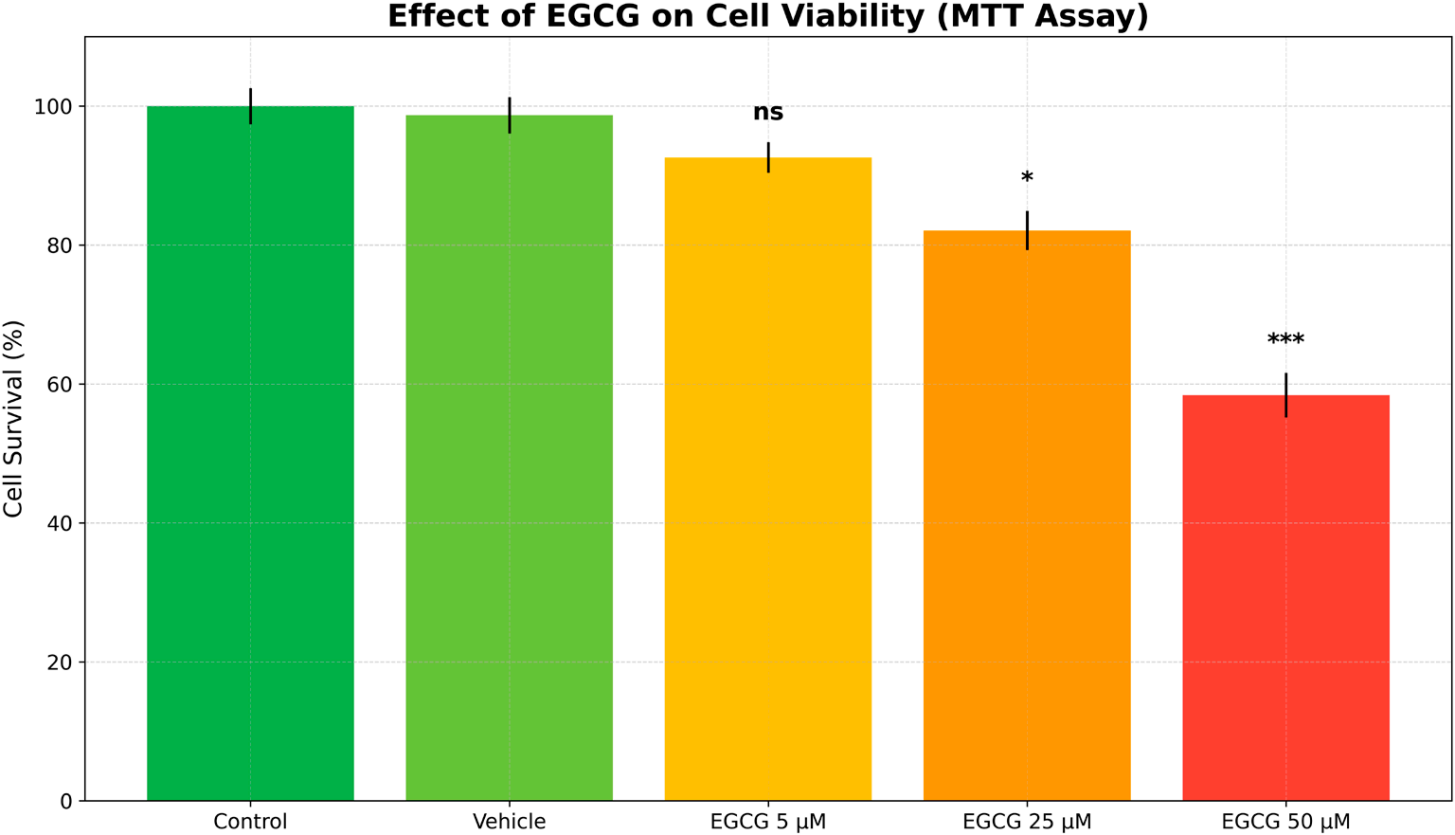
EGCG effect on cell viability (MTT assay).

At a concentration of 5 μM of EGCG, a slight decrease in cell survival (92.6%) was observed without statistical significance (ns), indicating that this concentration is relatively safe for cells. At a concentration of 25 μM, a greater decrease in cell survival (82.1%) occurred, which was statistically significant (*p* < 0.005), suggesting the onset of a dose‐dependent cytotoxic effect. The 50 μM concentration recorded a significant decrease in cell survival rate to 58.4%, which is highly statistically significant (*p* < 0.001), demonstrating a clear cytotoxic or pro‐apoptotic activity of EGCG at higher doses (all these findings were reported in Table 5 and Figure 3). These results were identical to those reported in the study of Fen et al. [17], Xuerui et al. [18], and Leonilla et al. [19], in which there was a decrease in cell survival rather than control.

**TABLE 5:** MTT viability results after 24 h treatment.

Group	EGCG (μM)	Cell survival (%) ± SD	Statistical significance
Control	0	100.0 ± 2.6	—
Vehicle	0	98.7 ± 2.6	ns
EGCG‐treated	5	92.6 ± 2.2	ns
EGCG‐treated	25	82.1 ± 2.8	*p* < 0.005
EGCG‐treated	50	58.4 ± 3.2	*p* < 0.001

### 3.3 Genes Expression

**FIGURE 4:**
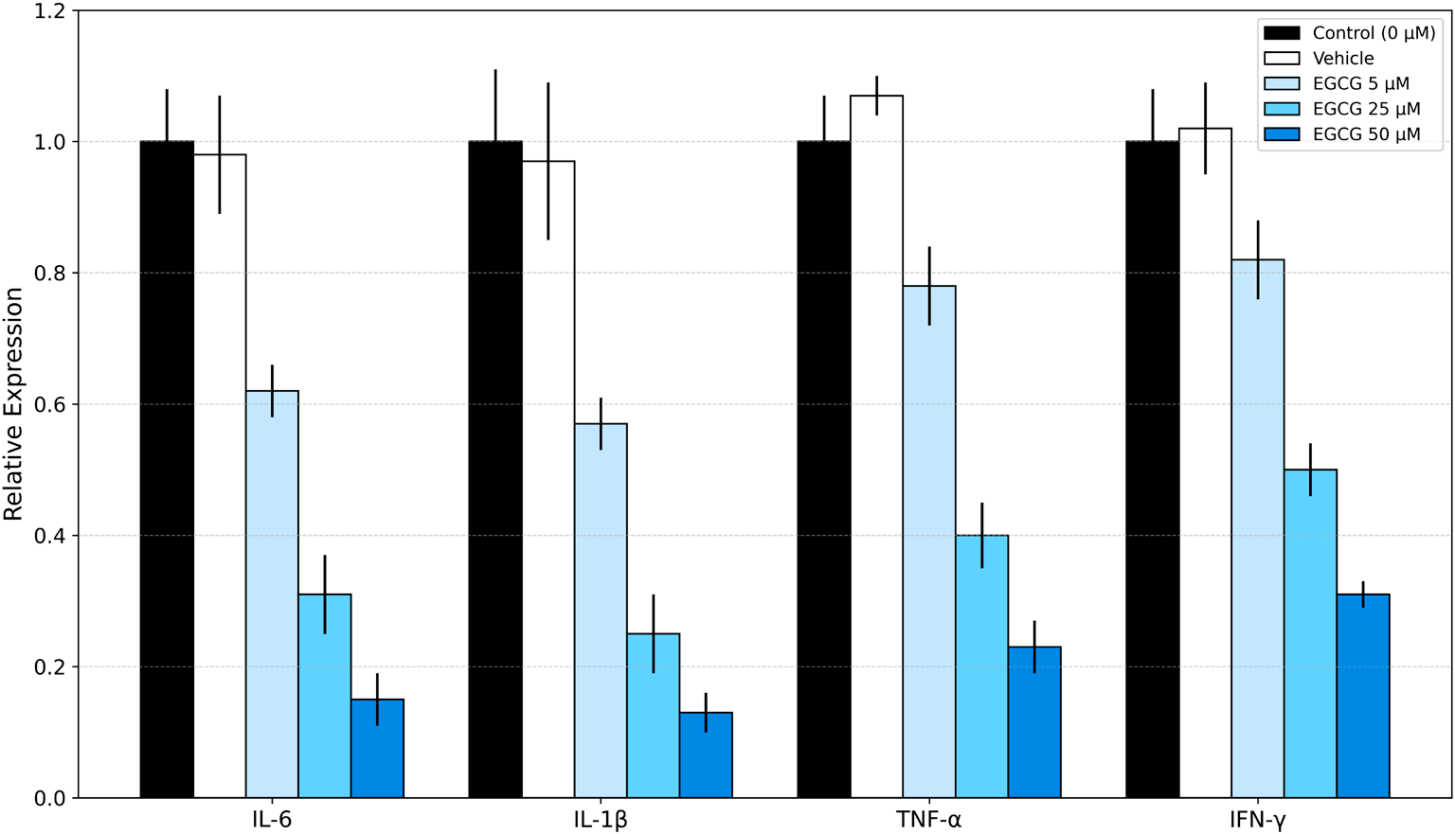
EGCG effect on pro‐inflammatory cytokine genes in peripheral blood mononuclear cells (PBMCs) of patients suffering from thalassemia.

EGCG showed a dose‐dependent inhibitory effect on the expression of the inflammatory genes IL‐6, IL‐1β, TNF‐α, and IFN‐γ in the blood cells of thalassemia patients (Table 6 and Figure 4). Gene expression at 5 μM was relatively low compared to the control and vehicle, but this difference was not statistically significant. With increasing doses (25 and 50 μM), expression showed a statistically significant decrease (*p* < 0.001 for IL‐6 and IL‐1β, *p* < 0.01 for TNF‐α, and *p* < 0.05 for IFN‐γ). IL‐6 and IL‐1β showed the greatest inhibition at 50 μM (0.15 ± 0.04 and 0.13 ± 0.03, respectively), suggesting a central role for them in inflammation associated with depression in patients with thalassemia. These observations mirror the results of Debora et al. [20], Nahid et al. [21], and BaoHe et al. [22].

**TABLE 6:** Fold change in mRNA expression levels of depression‐related cytokines by RT‐qPCR in PBMCs.

Target gene	Control (0 μM)	Vehicle	EGCG 5 μM	EGCG 25 μM	EGCG 50 μM	Statistical significance
IL‐6	1.00 ± 0.08	0.98 ± 0.09	0.62 ± 0.04	0.31 ± 0.06	0.15 ± 0.04	*p* < 0.001 (25 and 50 μM)
IL‐1β	1.00 ± 0.11	0.97 ± 0.12	0.57 ± 0.04	0.25 ± 0.06	0.13 ± 0.03	*p* < 0.001 (25 and 50 μM)
TNF‐α	1.00 ± 0.07	1.07 ± 0.03	0.78 ± 0.06	0.40 ± 0.05	0.23 ± 0.04	*p* < 0.01 (25 and 50 μM)
IFN‐γ	1.00 ± 0.08	1.02 ± 0.07	0.82 ± 0.06	0.50 ± 0.04	0.31 ± 0.02	*p* < 0.05 (25 and 50 μM)

The likeness of the control and vehicle values also demonstrates that the inhibition is produced by EGCG and not by the solvent compound. These findings support EGCG as a potential regulator of inflammatory gene expression in thalassemia.

### 3.4 ELISA Analysis

Significant reductions, in a dose‐dependent manner of the levels of cytokine proteins (IL‐6, IL‐1β, TNF‐α, and IFN‐γ) implicated in inflammation and linked to depression in thalassemia patients following EGCG treatment were confirmed by ELISA and RT‐qPCR data. EGCG, in doses of 25 and 50 μM, inhibited protein levels of these cytokine proteins, and these findings were consistent with the decreases in mRNA levels of the corresponding genes (Table 7). The agreement between ELISA and gene expression analyses suggests that the modulatory impact of EGCG leads to a reduction in cytokine secretion and a suppression of gene expression. The impact is especially evident for IL‐6 and IL‐1β, consistent with their established roles as central mediators in neuroinflammation and depression.

**TABLE 7:** ELISA analysis: cytokine suppression by EGCG.

Cytokine	Control (pg/mL)	Vehicle	EGCG 5 μM	EGCG 25 μM	EGCG 50 μM	Statistical significance
IL‐6	100 ± 7	98 ± 7	64 ± 8	32 ± 9	16 ± 5	*p* < 0.001 (25 and 50 μM)
IL‐1β	96 ± 9	95 ± 10	62 ± 8	27 ± 2	11 ± 1	*p* < 0.001 (25 and 50 μM)
TNF‐α	111 ± 5	117 ± 1	87 ± 2	42 ± 2	26 ± 6	*p* < 0.01 (25 and 50 μM)
IFN‐γ	107 ± 9	109 ± 3	82 ± 1	53 ± 5	31 ± 4	*p* < 0.05 (25 and 50 μM)

In contrast, while TNF‐α and IFN‐γ levels also showed significant reductions, their slightly higher residual levels may reflect their broader responsibilities in maintaining immune system equilibrium.

The reduction of cytokine production we observed in the present study seems to further suggest that EGCG might affect those inflammatory signaling cascades (such as NF‐κB) that directly upregulate the expression of these mediators. In addition, the observed patterns in ELISA were also accompanied by similar behaviors in RT‐qPCR and hence enhanced the credibility of the results and discarded the likelihood that these effects are just post‐transcriptional regulation.

Therefore, this investigation appears to provide evidence that EGCG could be used in combination to be used as an adjuvant treatment to manage neuroinflammatory symptoms in thalassemia patients, which might result in better mood‐related outcomes through cytokine modulation.

**FIGURE 5:**
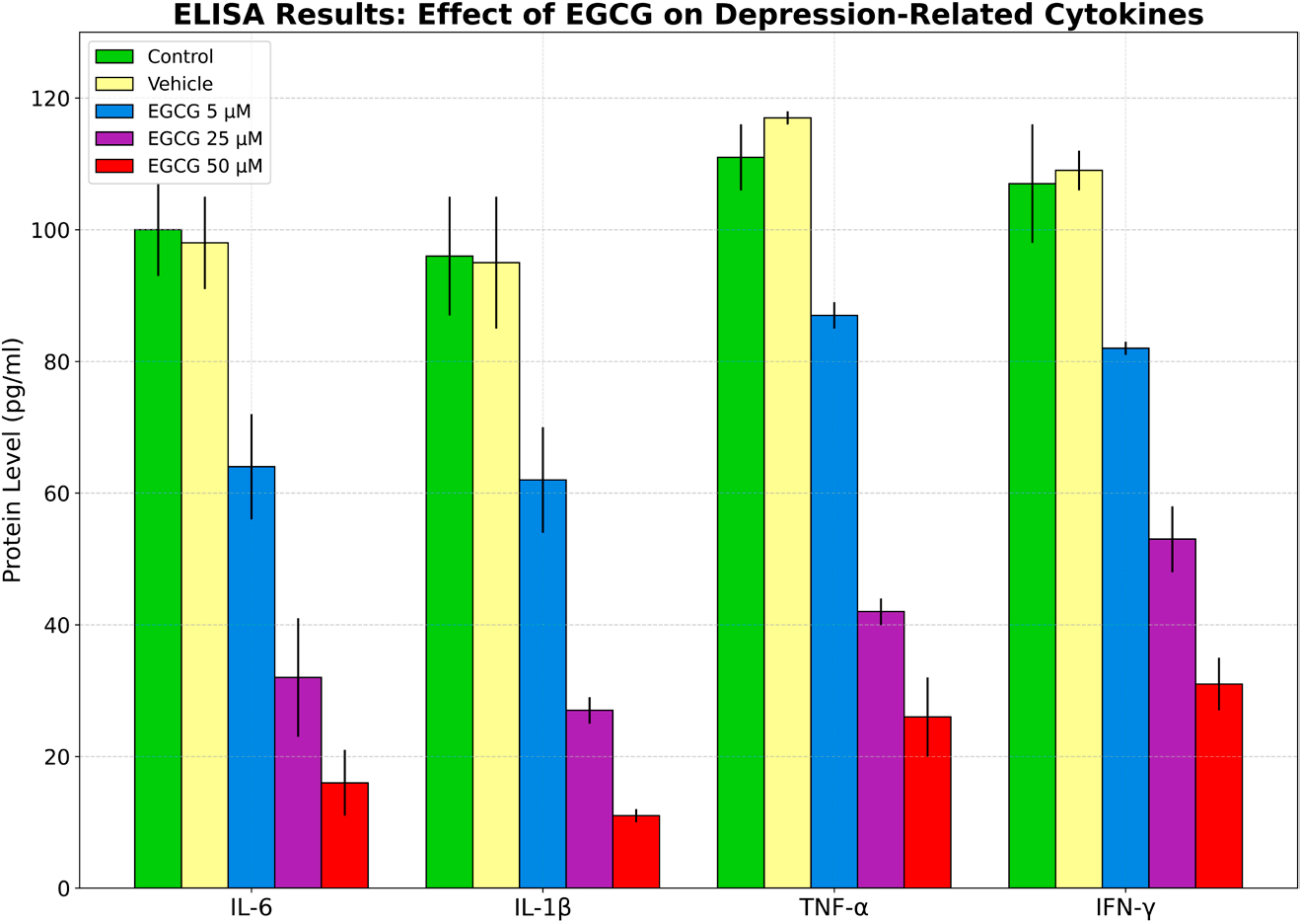
EGCG effect on depression‐related cytokines.

The graph in Figure 5 shows the effect of EGCG on depression‐related cytokines in thalassemia patients through ELISA test results, with each group representing a different color.

## Funding

The authors have nothing to report.

## Ethics Statement

This study was conducted in accordance with the Declaration of Helsinki and approved by the scientific committee of the Clinical Pharmacy Department/College of Pharmacy at Jabir Ibn Hayyan University for Medical and Pharmaceutical Sciences, Iraq. Approval was obtained from Al‐Najaf Health Directorate, Ministry of Health, and Information Center for Research & Development of Najaf Province. 3. The objectives and methodology were explained to all participants and verbal consent in the study had been taken.

## Conflicts of Interest

The authors declare no conflicts of interest.

## Data Availability

The data that support the findings of this study are available from the corresponding author upon reasonable request.

## References

[1] ACMC , A. L. E. de Oliveira , A. A. L. Rodrigues , et al., “Hematological Ratios and Cytokine Profiles in Heterozygous Beta‐Thalassemia,” Hematology, Transfusion and Cell Therapy 47, no. 3 (2025): 103845. 10.1016/j.htct.2025.103845 PMC12140038 40367897

[2] A. Dordevic , I. Mrakovcic‐Sutic , S. Pavlovic , M. Ugrin , and J. Roganovic , “Beta Thalassemia Syndromes: New Insights,” World Journal of Clinical Cases 13, no. 10 (2025): 100223. 40191679 10.12998/wjcc.v13.i10.100223 PMC11670029

[3] M. Faranoush , P. Faranoush , I. Heydari , et al., “Complications in Patients With Transfusion Dependent Thalassemia: A Descriptive Cross‐Sectional Study,” Health Science Reports 6, no. 10 (2023): e1624. 37841947 10.1002/hsr2.1624 PMC10568004

[4] L. Xie , J. Fang , J. Yu , W. Zhang , Z. He , and L. Ye , “The Role of CD4+ T Cells in Tumor and Chronic Viral Immune Responses,” MedComm (2020) 4, no. 5 (2023): e390. 37829505 10.1002/mco2.390 PMC10565399

[5] A. Sălcudean , C. R. Bodo , R. A. Popovici , et al., “Neuroinflammation. A Crucial Factor in the Pathophysiology of Depression: A Comprehensive Review,” Biomolecules 15, no. 4 (2025): 502. 10.3390/biom15040502 PMC12024626 40305200

[6] M. Menegazzi , R. Campagnari , M. Bertoldi , R. Crupi , R. Di Paola , and S. Cuzzocrea , “Protective Effect of Epigallocatechin‐3‐Gallate (EGCG) in Diseases With Uncontrolled Immune Activation: Could Such a Scenario Be Helpful to Counteract COVID‐19?,” International Journal of Molecular Sciences 21, no. 14 (2020): 5171. 32708322 10.3390/ijms21145171 PMC7404268

[7] L. Capasso , L. De Masi , C. Sirignano , et al., “Epigallocatechin Gallate (EGCG): Pharmacological Properties, Biological Activities and Therapeutic Potential,” Molecules 30, no. 3 (2025): 654. 39942757 10.3390/molecules30030654 PMC11821029

[8] P. Israelsson , E. Dehlin , I. Nagaev , E. Lundin , U. Ottander , and L. Mincheva‐Nilsson , “Cytokine mRNA and Protein Expression by Cell Cultures of Epithelial Ovarian Cancer, Methodological Considerations on the Choice of Analytical Method for Cytokine Analyses,” American Journal of Reproductive Immunology 84, no. 1 (2020): e13249. 32307767 10.1111/aji.13249

[9] J. B. Harborne , Phytochemical Methods: A Guide to Modern Techniques of Plant Analysis (Springer Science & Business Media, 1998).

[10] R. Amarowicz , F. Shahidi , and W. Wiczkowski , “Separation of Individual Catechins From Green Tea Using Silica Gel Column Chromatography and HPLC,” Journal of Food Lipids 10, no. 2 (2003): 165–177.

[11] A. Bhatia , T. Kaur , B. Singh , R. Arora , and S. Arora , “Reverse Phase HPLC Method Validation for Estimation of Polyphenols in Medicinal Plants and Their Possible Role in Reticence of Xanthine Oxidase Activity,” Separation Science Plus 2, no. 7 (2019): 237–244.

[12] E. Blahová and J. Lehotay , “Sample Preparation and HPLC Determination of Catechins in Green Tea,” Chemia Analityczna 51, no. 5 (2006): 795–808.

[13] T. B. Tra and T. L. P. Bui , “Development and Validation of a HPLC Method of Quantification of Caffeine and EGCG in Green Tea (*Camellia sinensis* L.) Extract,” VNU Journal of Science: Medical and Pharmaceutical Sciences 36, no. 3 (2020): 24–32.

[14] W. M. A. Niessen , Liquid Chromatography–Mass Spectrometry, Third Edition (CRC Press, 2006).

[15] T. Mosmann , “Rapid Colorimetric Assay for Cellular Growth and Survival: Application to Proliferation and Cytotoxicity Assays,” Journal of Immunological Methods 65, no. 1–2 (1983): 55–63. 6606682 10.1016/0022-1759(83)90303-4

[16] A. Boyum , “Isolation of Mononuclear Cells and Granulocytes From Human Blood. Isolation of Mononuclear Cells by One Centrifugation, and of Granulocytes by Combining Centrifugation and Sedimentation at 1g,” Scandinavian Journal of Clinical and Laboratory Investigation. Supplementum 97 (1968): 77–89. 4179068

[17] F. Wu , H. Sun , T. Kluz , C. Hailey , K. Kiok , and M. Costa , “Epigallocatechin‐3‐Gallate (EGCG) Protects Against Chromate‐Induced Toxicity *In Vitro* ,” Toxicology and Applied Pharmacology 258, no. 2 (2012): 166–175. 22079256 10.1016/j.taap.2011.10.018 PMC3259276

[18] X. Chen , B. Liu , R. Tong , et al., “Improved Stability and Targeted Cytotoxicity of Epigallocatechin‐3‐Gallate Palmitate for Anticancer Therapy,” Langmuir 37, no. 2 (2021): 969–977. 33393784 10.1021/acs.langmuir.0c03449

[19] L. Elbling , R.‐M. Weiss , O. Teufelhofer , et al., “Green Tea Extract and (−)‐Epigallocatechin‐3‐Gallate, the Major Tea Catechin, Exert Oxidant but Lack Antioxidant Activities,” FASEB Journal 19, no. 7 (2005): 1–26. 15738004 10.1096/fj.04-2915fje

[20] D. Porath , C. Riegger , J. Drewe , and J. Schwager , “Epigallocatechin‐3‐Gallate Impairs Chemokine Production in Human Colon Epithelial Cell Lines,” Journal of Pharmacology and Experimental Therapeutics 315, no. 3 (2005): 1172–1180. 16123309 10.1124/jpet.105.090167

[21] N. Akhtar and T. M. Haqqi , “Epigallocatechin‐3‐Gallate Suppresses the Interleukin‐1‐Beta Induced Inflammatory Response in Human Chondrocytes,” Arthritis Research & Therapy V13 (2011): R93. 10.1186/ar3368 PMC3218908 21682898

[22] B.‐H. Zhu , H.‐Y. Chen , W.‐H. Zhan , et al., “Epigallocatechin‐3‐Gallate Inhibits VEGF Expression Induced by IL‐6 *via* Stat3 in Gastric Cancer,” World Journal of Gastroenterology 17, no. 18 (2011): 2315–2325. 21633597 10.3748/wjg.v17.i18.2315 PMC3098399

